# Medical Students' Perspectives on Physical, Online, and Hybrid Learning Modalities: A Mixed Methods Study From a Medical School in Mauritius

**DOI:** 10.7759/cureus.82765

**Published:** 2025-04-22

**Authors:** Jared Robinson, Indrajit Banerjee, Driti Reechaye, Anne Laure A Perrine

**Affiliations:** 1 Surgery, Sir Seewoosagur Ramgoolam Medical College, Vacoas-Phoenix, MUS; 2 Pharmacology, Sir Seewoosagur Ramgoolam Medical College, Vacoas-Phoenix, MUS; 3 Internal Medicine, Sir Seewoosagur Ramgoolam Medical College, Vacoas-Phoenix, MUS

**Keywords:** adult and tertiary teaching, education, medical education, online medical education, online teaching, teaching, virtual teaching

## Abstract

Introduction: Medical schools were not immune to the difficulties posed by the COVID pandemic. Educational institutions were shut down. By April 2020, 73.8% of registered students in 186 countries were adversely affected. The circumstances forced academic institutions to transition to emergency online teaching, despite the controversies of the effectiveness of this method. The absence of face-to-face teaching greatly impacted the clinical placements and affected the physical and practical skills training of many institutions.

Objective: There is a dearth of data showing how medical students perceive blended learning. As far as the authors are aware, no study has ever addressed the experience of medical students regarding blended, online, and physical teaching in Mauritius. Therefore, the objective of the research is to address the challenges they faced and provide suggestions to tackle the drawbacks and help the betterment of the style of teaching with enhanced academic performance. A qualitative and quantitative assessment of the students’ experience of these three methods was conducted.

Methods: A mixed methods study, incorporating both qualitative and quantitative data, was conducted at Sir Seewoosagur Ramgoolam Medical College, Mauritius, to understand medical students' perceptions of online, hybrid, and physical teaching. Statistical Product and Service Solutions (SPSS, version 29; IBM SPSS Statistics for Windows, Armonk, NY) was used for quantitative data analysis, whereas NVivo 12 Plus software was used for qualitative data analysis.

Results: Out of 700 participants, 568 medical students participated in the study for quantitative data synthesis, resulting in an overall response rate of 81.14% (568). A total of 224 (69.1%) female students preferred a mixture of online and in-person lectures, followed by didactic lectures (100, 30.9%) and online (0, 0%). Among all male students, 160 (65.6%) preferred a mixture of online and in-person lectures, didactic lectures (68, 27.5%), and online lectures (16, 6.6%) (p<0.05). Webex was the most popular online video conferencing application utilized for the lectures, which was preferred by 248 (76.5%) female students, followed by Zoom (52, 16%), Google Meet (20, 6.2%), and Microsoft Teams (4, 1.2%). Regarding male students, 200 (82%) students preferred Webex, Zoom (32, 13.1%), Microsoft Teams (8, 3.3%), and Google Meet (4, 1.6%), which was statistically significant (p<0.05). A total of 144 (77.8%) Mauritian students, 34 (73.9%) South Africans, and 206 (61.7%) Indian students preferred a mixture of online and in-person lectures (p<0.05). A descriptive phenomenological qualitative data analysis generated three themes from the codes were as follows: online learning system, physical learning system, and blended learning system. Each theme had three different categories - emotional, academic, and related variables. Under each category, related codes were organized.

Conclusion: The novelty of this study is evident, and its practical implications stretch far beyond the lockdown and COVID-19, but its findings, particularly surrounding the reduction in the loss of class times during tropical storms and hurricanes, are invaluable. The hybrid model of teaching caters to a wider audience of students and helps create a balanced mental and physical educational experience, resulting in the best outcome for students in their studies by capitalizing on the advantages of both didactic and online lectures.

## Introduction

Once the World Health Organization (WHO) declared the novel coronavirus disease 2019 (COVID-19) a global pandemic on the 11th of March 2020, the disease has posed challenges to every sector of the economy and education. Medical schools were not immune to the difficulties posed by the pandemic [1]. Owing to the fact that the virus spreads via droplets, one of the major preventive measures was social distancing, which led to national lockdowns. Educational institutions were shut down, and, by April 2020, 73.8% of registered students in 186 countries were adversely affected [2].

As for Mauritius, the initial closure of educational institutions lasted from the 20th of March to the 1st of July 2020, which was an immediate response to the detection of the first three cases of COVID-19 on the island on the 18th of March [3]. Online teaching placed strain on both medical students and lecturers, as medical education is patient-centered. Medical schools were not adequately prepared to face such a rapid transition [4]. The speed of the transition and the fear induced by the pandemic had impacts on the mental health, as well as the physical health, of students and lecturers. Lecturers were not always adequately trained to operate online teaching platforms, which created difficulty. The absence of face-to-face teaching greatly impacted the clinical placements and affected the physical practical skills training.

Developing nations had a lack of uniformity in the availability and access to good-quality technological tools and internet connections among both students and lecturers [3]. On the other hand, e-learning provided the convenience of attending classes from the comfort of one’s home with more flexible timing and accessibility of class materials.

As for the staff and students, anti-coronavirus preventive measures, the introduction of vaccinations, staggered time tables, and a hybrid type of learning known as blended learning became the norm [3]. Blended learning is described as a combination of traditional face-to-face learning and asynchronous or synchronous e-learning. This manner of learning and education has experienced a rise in popularity as its implementation and usefulness extend beyond the COVID-19 pandemic and may be the solution in case of other major challenges faced by institutions [5]. The integration of technology, even in the post-pandemic era, may be helpful in the acquisition of practical skills for students and lecturers alike [6]. As the traditional in-class teaching is implemented in conjunction with online, their strengths act synergistically. However, the effectiveness of this method of teaching is greatly influenced by the level of technological progress and availability of necessary standard tools from the institution [4].

Though we are in a technological era, there is a dearth of data showing how medical students perceive blended learning. As far as the authors are aware, no study has ever addressed the experience of medical students with regard to blended, online, and in physical teaching in Mauritius. Therefore, the aim of the research is to address the challenges they faced and put forward their suggestions to tackle the drawbacks and hence help the betterment of the style of teaching with enhanced academic performance, in the future. Additionally, a qualitative and quantitative assessment of the students’ experience of these three methods was conducted.

## Materials and methods

Study design and participants

A mixed method study was conducted at Sir Seewoosagur Ramgoolam Medical College, Mauritius. Out of 700 participants, 568 medical students participated in the study for quantitative data synthesis, resulting in an overall response rate of 81.14 %, 324 of these students being female and 224 being male. A total of 334 of these students were Indian, 185 Mauritian, and 46 South African.

A phenomenological qualitative data analysis was conducted by one-on-one interviews among 11 students. Participants from all the levels of study, both males and females, and of different nationalities (Indians, Mauritians, and South Africans) were included. Selection of participants for the qualitative study was done using the convenience sampling method. Participants selected were then approached in person, and the interview was conducted face to face, on the school premises. None of the 11 students approached for the interview refused or dropped out of the study, and no repeat interviews had to be conducted.

Data collection

Quantitative data were collected between the 1st of September 2023 to the 15th of January 2024. Data were collected using Google Forms, which was distributed to all the students who were pursuing a medical degree at Sir Seewoosagur Ramgoolam Medical College, Mauritius. Informed consent was obtained from every participant before taking part in the survey.

For the qualitative data, in-depth interviews were conducted by the authors, and interviews were recorded until data saturation occurred and, on average, were 15 minutes in duration. The interview data were transcribed by the authors using both voice-to-text translation applications and manual transcription methods. The interviews were held in a one-on-one private setting with the oversight and guidance of the senior author. To maintain privacy and confidentiality, reporting was done anonymously, and recorded data were stored securely to prevent disclosure of the data. The data collected were used only for the purpose of this study. Furthermore, the interviewers in no way had a conflict of interest with the interviewees, as they were not involved in examining the students. Anonymity of the students and their answers was also maintained throughout the study process.

Questionnaire design

After an extensive review of the literature, a questionnaire was developed so that they were relevant to meet the objective of the study. The final questionnaire was divided into three sections: demographic details, the quantitative section - hybrid, online classes, and physical didactic lectures - and the qualitative section.

Questionnaire validity

A panel of four subject experts consisted of a public health officer, an academician, a pharmacologist, and a medical educationist. The content and construct validity were assessed by the four experts. The questionnaire was modified based on their comments. The questionnaire obtained an average congruency percentage of 90%.

Face validity

A pilot study was conducted among eight students from all the batches for readability and understanding of the questionnaire. Based on their recommendation, the questionnaire was modified. The revised questionnaire was applied for the primary data collection.

Reliability analysis

Cronbach's alpha test was used for the reliability analysis of the quantitative part of the questionnaire. The internal consistency between the items using Cronbach's alpha was found to be 0.72.

Inclusion criteria

All the medical students who were pursuing a Bachelor of Medicine, Bachelor of Surgery (MBBS) degree at Sir Seewoosagur Ramgoolam Medical College, Mauritius, were included in the study; 568 out of 700 participated in this study for the quantitative data analysis. Eleven students participated in the qualitative data analysis, ensuring that all of the years of study were included in the qualitative aspect of the study.

Exclusion criteria

Students who had not given their consent to participate in the study were excluded from the study.

Outcome variable

The main outcome variables of the study were the perception of medical students on hybrid, online classes, and physical didactic lectures.

Explanatory variable

Factors that were considered on the individual level were gender (male and female), nationality (Mauritian, Indian, South African, and others), religion (Hinduism Muslim, Christian, and Atheism), level of study (first professional, second professional, final part p Professional, and final part II professional).

Ethical clearance

Prior to the study, ethical clearance was obtained from the Ethics and Research Committee, Ministry of Education, Tertiary Education, Science and Technology, Government of Mauritius (Approval Code- MTE/CR/213 V3), on 12 April 2023.

The research was subsequently approved to be conducted in accordance to the latest version of the Declaration of Helsinki - Ethical Principles for Medical Research involving Human Subjects guidelines.

Sample size calculation

Sample size calculation was done prior to the data collection using OpenEpi, a web-based program [7]. The sample size was estimated to be 245, calculated at the 95% confidence interval. The population size used for for finite population correction factor (FPC) was N=1000. The hypothesized percentage frequency of outcome factor in the population (p) was 70%, with a margin of error +/-5. The confidence limits (d) were set at an absolute +/- 5%. A design effect (DEFF) of 1 was applied, considering that it was a cluster survey. We achieved an adequate sample size of 568 for the quantitative data analysis:

$\displaystyle{\displaylines{n = \frac{\text{DEFF} \times N p (1 - p)}{\frac{d^2}{Z^2_{1-\alpha/2}} (N - 1) + p (1 - p)}}}$

Qualitative data were collected following the guidelines of Glaser et al. until data saturation was achieved, meaning no new codes/nodes were generated upon interview [8].

Data management and statistical analysis

The quantitative data were managed and analyzed using the Statistical Product and Service Solutions (SPSS, version 29; IBM SPSS Statistics for Windows, Armonk, NY). The chi-squared test was used to establish the statistical association between variables, and p < 0.05 was considered statistically significant.

The qualitative data were transcribed by the authors (DR, ALAP). NVivo 12 (Windows) Plus software (QSR International, Melbourne, Australia) was used to generate the nodes/codes and the various themes. The data were analyzed in accordance to Braun and Clarke’s Six Phases of Thematic Analysis [9].

The six phases of the thematic analysis included the following: (1) familiarization with the data, where the authors read the data and transcripts and noted initial ideas and observations; (2) generation of the initial codes through systematic identification and labelling was performed; (3) themes were identified via the collation and grouping of the specific nodes and data generated; (4) the themes, in relation to the extracted codes, were reviewed and further refined; (5) defining and naming the specific identified themes were done; and (6) through specific examples, the final analysis was done, and the findings of the study were reported.

## Results

Quantitative data

The mean age was found to be 21.76 ±1.641 SD years. Table 1 depicts that the majority of students who partook in the study were Indian (334, 58.8%), Mauritian (185, 32.6%), and South African (46, 8.1%) nationals. As far as the religion of students was concerned, the majority were Hindus (440, 77.5%), Muslims (59, 10.4%), and Christians (52, 9.2%), with atheism only accounting for 17 (3%) of the study population.

**Table 1:** Demographic details of students and teaching and learning methods (frequency n (%), total n= 568) ** P value: less than 0.05 (statistically significant); x P value: more than 0.05 (statistically insignificant) MBBS: Bachelor of Medicine, Bachelor of Surgery

Demographic profile		Female	Male	Total	P value
Nationality	Indian	193 (59.6)	141 (57.8)	n=334 (58.8)	0.350^X^
	Mauritian	100 (30.9)	85 (34.8)	n=185 (32.6)	
	South African	28 (8.6)	18 (7.4)	n=46 (8.1)	
	Others	3 (0.9)	0 (0)	n=3(0.5)	
Religion	Hindu	268 (82.7)	172 (70.5)	n=440 (77.5)	0.000**
	Muslim	17 (5.2)	42 (17.2)	n=59 (10.4)	
	Christian	34 (10.5)	18 (7.4)	n=52 (9.2)	
	Atheist	5 (1.5)	12 (4.9)	n=17 (3)	
Level of study	1^st^ MBBS	81 (25)	57 (23.4)	n=138 (24.3)	0.969^X^
	2^nd^ MBBS	94 (29)	74 (30.3)	n=168 (29.6)	
	Final Part 1	88 (27.2)	66 (27)	n=154 (27.1)	
	Final Part 2	61 (18.8)	47 (19.3)	n=108 (19)	
Preclinical/Clinical	Preclinical	175 (54)	131(53.7)	n=306 (53.9)	0.503^x^
	Clinical	149 (46)	113 (46.3)	n=262 (46.1)	
Experienced online teaching during the COVID-19 pandemic/rainy days or any other reason	Yes	316 (97.5)	232 (95.1)	n=548 (96.5)	0.09^X^
	No	8 (2.5)	12 (4.9)	n=20 (3.5)	
Duration of online classes attended	Less than 1 month	88 (27.2)	52 (21.3)	n=140 (24.6)	0.242^X^
	1 month	12 (3.7)	16 (6.6)	n=28 (4.9)	
	3 months	48 (14.8)	44 (18)	n=92 (16.2)	
	4 months	8 (2.5)	8 (3.3)	1n=6 (2.8)	
	6 months	168 (51.9)	124 (50.8)	n=292 (51.4)	

Among all the female students, most of them were Hindus (268, 82.7%), followed by Christians (34, 10.5%), Muslims (17, 5.2%), and atheists (only accounting for five, 1.5%). Meanwhile, among the male students, the majority of students were Hindus (172, 70.5%), followed by Muslims (42, 17.2%), Christians (18, 7.4%), and atheists (accounting for only 12, 4.9%). This finding was found to be statistically significant (p<0.05).

A total of 168 (29.2%) students were in their second year of their MBBS training, the majority of them being preclinical in training (306, 53.9%). A cohort of 384 (67.6%) preferred blended learning, and 548 (96.5%) were exposed to online methods of teaching. The vast majority of students experienced such online lectures for a period of six months (292, 51.4%).

Table 2 depicts that, among all the female students, 224 (69.1%) preferred a mixture of online and in-person lectures, followed by didactic lectures (100, 30.9%) and online (0, 0%). Meanwhile, among all male students, 160 (65.6%) preferred a mixture of online and in-person lectures, followed by didactic lectures (68, 27.5%) and online (16, 6.6%). This finding was found to be statistically significant (p<0.05).

**Table 2:** Correlation between demographic details and experienced online classes during the COVID-19 outbreak/rainy days, preference to teaching and learning, and online platform used ** P value: less than 0.05 (statistically significant); x P value: more than 0.05 (statistically insignificant)

Demographic profile		Experienced online classes during COVID-19 outbreak/rainy days		Which teaching and learning method do you prefer?			Online platform used				Can online lectures when better built into a timetable better optimize your studying	
Response		No	Yes	A mixture of a blend of online and in-person	Didactic lecture (in person)	Online	Google meet	Microsoft teams	Webex	Zoom	No	Yes
Gender	Female	8 (2.5)	316 (97.5)	224 (69.1)	100 (30.9)	0 (0)	20 (6.2)	4 (1.2)	248(76.5)	52 (16)	24 (7.4)	300 (92.6)
	Male	12 (4.9)	232 (95.1)	160 (65.6)	68 (27.9)	16 (6.6)	4 (1.6)	8 (3.3)	200 (82)	32 (13.1)	20 (8.2)	224 (91.8)
P value		0.117^X^		0.000**			0.013**				0.728^x^	
Nationality	Indian	16 (4.8)	318 (95.2%)	206 (61.7)	112 (33.5)	16 (4.8)	20 (6)	5 (1.5)	254 (76)	55 (16.5)	32 (9.6)	302 (90.4)
	Mauritian	0 (0)	185 (100)	144 (77.8)	41 (22.2)	0 (0)	4 (2.2)	3( 1.6)	153 (82.7)	25 (13.5)	8 (4.3)	177 (95.7)
	South African	4 (8.7)	42 (91.3)	34 (73.9)	12 (26.1)	0 (0)	0 (0)	4 (8.7)	38 (82.6)	4 (8.7)	4 (8.7)	42 (91.3)
	Others	0 (0)	3 (100)	0 (0)	3 (100)	0( 0)	0 (0)	0 (0)	3 (100)	0 (0)	0 (0)	3 (100)
P value		0.007**		0.000**			0.017**				0.178^x^	
Preclinical/Clinical	Clinical	4 (1.5)	258 (98.5)	175 (66.8)	76 (29)	11 (4.2)	11 (4.2)	3 (1.1)	226 (86.3)	22 (8.4)	13 (5)	249 (95)
	Preclinical	16 (5.2)	290 (94.8)	209 (68.3)	92 (30.1)	5 (1.6)	13 (4.2)	9 (2.9)	222 (72.5)	62 (20.3)	31 (10.1)	275 (89.9)
P value		0.021**		0.183^X^			0.000**				0.027**	

Webex was the most popular online video conferencing application utilized for the lectures, which was preferred by 248 (76.5%) female students, followed by Zoom (52, 16%), Google Meet (20, 6.2%), and Microsoft Teams (4, 1.2%). As far as male students are concerned, 200 (82) students preferred Webex, followed by Zoom (32, 13.1%), Microsoft Teams (8, 3.3%), and Google Meet (4, 1.6%). This was found to be statistically significant (p<0.05).

The following preferred a mixture of online and in-person lectures: 144 (77.8%) of the Mauritian students, 34 (73.9%) of the South African students, and 206 (61.7%) of the Indian students (p<0.005). The following preferred Webex as an ideal platform: 254 (76%) of all the Indian students, 153 (82.7%) of all the Mauritian students, and 38 (82.6%) South African students. This finding was found to be statistically significant (p<0.05).

The following agreed with the statement that online lectures, when better built into a timetable, will better optimize your study: 249 (95%) clinical students and 275 (89.9%) preclinical students, whereas only 13 (5%) clinical and 31 (10.1%) preclinical students disagreed with this statement (p<0.05).

Table 3 depicts that 208 (64.2%) female students and 118 (44.3%) male students agreed that self-directed study was an advantage provided by online lectures, followed by ease of accessibility and timing (108 (33.3%) female and 120 (49.2%) male students) and enhanced lecture emphasis (8 (2.5%) female and 16 (6.6%) male students). This finding was found to be statistically significant (p<0.05).

**Table 3:** Association between demographic details/strengths and weakness and improvement required in online mode ** P value: less than 0.05 (statistically significant); x P value: more than 0.05 (statistically insignificant)

Demographic profile		Strengths of online lectures			Weakness of online lectures					Improvement required in online mode				
Response		Ease of accessibility and timing	Enhanced lecture emphasis	More self-directed study time	Distractions	Lack of accountability	Lack of interaction	Platforms used	Lack of student attention	Better preparation	Class engagement	Content	Internet	Timing
Gender	Female	108 (33.3)	8 (2.5)	208 (64.2)	108 (33.3)	20 (6.2)	84 (25.9)	64 (19.8)	48 (14.8)	76 (23.5)	112 (34.6)	48 (14.8)	60 (18.5)	28 (8.6)
	Male	120 (49.2)	16 (6.6)	108 (44.3)	68 (27.9)	32 (13.1)	32 (13.1)	64 (26.2)	48 (19.7)	52 (21.3)	72 (29.5)	40 (16.4)	36 (14.8)	44 (18)
P value		0.000**			0.000**					0.014**				
Nationality	Indian	156 (46.7)	13 (3.9)	165 (49.4)	104 (31.1)	29 (8.7)	74 (22.2)	74 (22.2)	53 (15.9)	59 (17.7)	140 (41.9)	46 (13.8)	54 (16.2)	35 (10.5)
	Mauritian	59 (31.9)	11 (5.9)	115 (62.2)	56 (30.3)	19 (10.3)	36 (19.5)	35 (18.9)	39 (21.1)	65 (35.1)	32 (17.3)	32 ( 17.3)	31 (16.8)	25 (13.5)
	South African	10 (21.7)	0 (0)	36 (78.3)	16 (34.8)	4 (8.7)	6 (13)	16 (34.8)	4 (8.7)	4 (8.7)	12 (26.1)	10 (21.7)	8 (17.4)	12 (26.1)
	Others	3 (100)	0 (0)	0 (0)	0 (0)	0 (0)	0( 0)	3 (100)	0 (0)	0 (0)	0 (0)	0 (0)	3 (100)	0 (0)
P value		0.000**			0.053^x^					0.000**				
Preclinical/Clinical	Clinical	87 (33.2)	17 (6.5)	158 (60.3)	85 (32.4)	30 (11.5)	47 (17.9)	52 (19.8)	48(18.3)	66 (25.2)	71 (27.1)	39 (14.9)	47 (17.9)	39 (14.9)
	Preclinical	141 (46.1)	7 (2.3)	158 (51.6)	91 (29.7)	22 (7.2)	69 (22.5)	76 (24.8)	48 (15.7)	62 (20.3)	113 (36.9)	49 (16)	49 (16)	33 (10.8)
P value		0.001**			0.183^X^					0.09^X^				

The major issues identified with the online lectures were distractions (108 (33.3%) females and 68 (27.9%) males), followed by the platform used/technological issues (64 (26.2%) males and 64 (19.8%) females). This finding was found to be statistically significant (p<0.05).

Class engagement was identified as the most common area for improvement (72 (29.5%) male and 112 (34.6%) female students), followed by better preparation (76 (23.5%) female and 52 (21.3%) male students). This finding was found to be statistically significant (p<0.05).

A total of 140 (41.9%) Indian students agreed that class engagement within online lectures can be improved, whereas only 32 (17.3%) of Mauritian and 12 (26.1%) South African students agreed with the notion that online class engagement should be improved (p<0.05).

Table 4 signifies that 176 (54.3%) female and 108 (44.3%) male students believed that online lectures affected them negatively (p<0.05).

**Table 4:** Correlation between demographic details: negativity of online lectures, frequency of accepted online lectures and hybrid classes can be a part of the syllabus ** P value: less than 0.05 (statistically significant); x P value: more than 0.05 (statistically insignificant)

Demographic details		Online lectures affected you negatively		How often would online lectures be acceptable and useful				Can hybrid classes be part of the syllabus	
Response		No	Yes	More than once weekly	Never	Once weekly	Only rainy days	No	Yes
Gender	Female	148 (45.7)	176 (54.3)	124 (38.3)	16 (4.9)	136 (42)	48 (14.8)	56 (17.3)	268 (82.7)
	Male	136 (55.7)	108 (44.3)	140 (54.7)	8 (3.3)	40 (16.4)	56 (23)	52 (21.3)	192 (78.7)
P value		0.018**		0.000**					0.226^x^
Nationality	Indian	162 (48.5)	172 (51.5)	137 (41)	13 (3.9)	112 (33.5)	72 (21.6)	72 (21.6)	262 (78.4)
	Mauritian	90 (48.6)	95 (51.4)	94 (50.8)	11 (5.9)	60 (32.4)	20 (10.8)	29 (15.7)	156 (84.3)
	South African	32 (69.6)	14 (30.4)	30 (65.2)	0 (0)	4 (8.7)	12 (26.1)	4 (8.7)	42 (91.3)
	Others	0 (0)	3 (100)	3 (100)	0 (0)	0( 0)	0 (0)	3 (100)	0 (0)
P value		0.015**		0.000**				0.000**	
Preclinical/Clinical	Clinical	132 (50.4)	130 (49.6)	134 (51.1)	10 (3.8)	83 (31.7)	50 (19.1)	212 (80.9)	66 (25.2)
	Preclinical	152 (49.7)	130 (42.5)	14 (4.6)	93 (30.4)	69 (22.5)	58 (19)	248 (81)	62 (20.3)
P value		0.886^x^		0.029**				0.09^X^	

This statement is balanced by the fact that 136 (42%) females and 40 (16.4%) males agreed that online lectures would be acceptable and useful once weekly. This is seconded by the use thereof of females (more than once weekly (124, 38.3%), only rainy days (48, 14.8%), and never (16, 4.9%)) and males (40, 16.4%; p<0.05).

The following agreed that hybrid classes can be part of the syllabus: 268 (82.7%) females and 192 (78.7%) males. Meanwhile, 156 (84.3%) of Mauritian and 42 (91.3%) of South African students agreed with this statement (p<0.05).

Clinical students agreed that online lectures would be acceptable: more than once weekly (134, 51.1%), useful once weekly (83, 31.7%), only on rainy days (50, 19.1%), and never (10, 3.8%). Preclinical medical students agreed that online lectures would never be acceptable (93, 30.4%), followed by once weekly (69, 22.5%), only on rainy days (58, 19%), and more than once weekly (14, 4.6%; p<0.05).

Qualitative data

Eleven students partook in the qualitative section of the study, seven of them being female and four being male (three South Africans, four Mauritians, and four Indians). Two of the students were in their first year, three of them were in their second MBBS, three in their final part 1, and three in their final part 2. 

An inductive thematic analysis was performed. Three themes were generated from the codes as follows: online learning system, physical learning system, and the blended learning system. Each theme had three different categories: emotional, academic, and related variables. Under each category, related codes were organized in Table 5.

**Table 5:** Phenomenological qualitative thematic analysis

Themes	Categories	Codes
Online learning system	Emotional	Stressed
		Mixed feelings
		Indifference
		Hesitant
		Difficult
	Academic	Flexibility
		Improvement
		Productive
		Boring
		Revision
	Related variables	No alternative
		Judicious use
		Internet connection
Physical learning system	Emotional	Depressed
		Exhaustive
		Burn out
		Predominant mood
	Academic	Experience
		Productivity
		Discipline
		Cumbersome schedule
		Hectic
		Self- directed learning
		Lecture delivery
	Related variables	Mental health
		Life balance
		Physical health
		Time consuming
Blended learning system	Emotional	Knowledgeable
	Academic	Efficiency
		Efforts
		Manageable
		Class material availability
		Active learning
		Interaction
		Better approach
	Related variables	Personal time
		Life balance
		Time saving
		Sedentary lifestyle
		Physical well-being
		Technological era

Various codes/nodes and the narratives are depicted in Table 6. The codes/nodes generated were as follows: difficult, stress, flexibility, mixed feelings, improvement, indifference, productive, boring, laziness, no alternative, judicious use, experience, lecture delivery, discipline, exhausting, time consuming, mental health, hectic, manageable, hesitant, life-balance, self-directed learning, efficient, efforts, personal time, time-saving, class material availability, revision, active learning, interaction, better approach, sedentary lifestyle, physical well-being, knowledge and confidence, burn out, internet connection, predominant mood, and technological era (Table 6).

**Table 6:** Codes/nodes generated

Codes/Nodes	Description	Narrative
Difficult	An act that requires a lot of effort to understand (e.g. online classes) or skills to accomplish (e.g. online teaching).	“Online classes were difficult to understand.” (S3, S5) “It was really difficult for teachers to use whiteboards/blackboards to teach. It was difficult to understand by powerpoints only.” (S5)
Stress	An increased mental pressure results from the online teaching experience.	“It was very stressful for me” (S7)
Flexibility	The ability to have more freedom and the capacity to accommodate a better study schedule.	“Online classes are good because they give us more flexibility.” (S5)
Mixed feeling	A state of having both negative and positive opinions in regards to the students’ online experience.	“I had mixed feelings.” (S10) “It was not good, not bad.” (S5)
Improvement	It was a newly introduced study design in our school and could have been ameliorated.	“Teachers need to get used to it and know how to carry online classes.” (S7) “I think there is scope for improvement.” (S4)
Indifference	The online classes neither brought any major change nor were they of much use.	“It really did not make much of a difference in me.” (S8)
Productive	Ability to gain maximum knowledge and understand the lecture.	Online: “I don't feel they are as productive as in person classes..” (S2). “I am more productive at home.” (S10)
Boring	The student found the online classes to be dull and tiresome.	“It would really make lives more boring than it already is.” (S9). “In online classes I was bored.” (S5)
Laziness	The state of unwillingness to show interest in a particular subject and work towards it.	Online: “It would make us into lazy students again.” (S3)
No alternative	Online classes were the only option to carry on with the syllabus.	“I don’t think they had any other option, the online teaching was the only option.” (S9). “It is fine to have online classes because we don't have any other option.” (S8)
Judicious use	Topics of selective subjects to be taught online should be wisely chosen before being incorporated in the schedule.	“It actually depends on subject to subject, in some classes where we only take notes, online can work but practical classes, only in physical would work.” (S6)
Experience	The opportunity to observe and practice different procedures and acquire related skills.	“We really need to go to hospital, interact with the patients, and the environment..” (S6) “We need to see the patients… slides or PowerPoint then we ourselves cannot understand as long as we see the things under the microscope, the patient.” (S4) “We have more hands- on experience in postings.” (S11)
Lecture delivery	The way in which the teachers teach classes in person is much better and more efficient.	“The teachers go at a better pace.” (S7) “Teachers can explain more to us and can see who is following.” (S5)
Discipline	A set of established rules and a schedule to which everyone is inclined to abide by.	“There is like a timetable, there is more discipline here, you know when the classes are starting, when finishing.” (S1)
Exhausting	A state of physical and mental tiredness that is experienced by the student in a particular setting.	BLENDED: “You will not be tired to revise.” (S5)
		ONLINE: “It is less tiring.” (S5)
		IN PHYSICAL: “Most of us are already tired by the time we reach home.” (S5)
Time consuming	A significant amount of time is spent getting prepared and traveling to college to attend physical classes.	“Time consuming to come here, for example, I use 2 to 2.5 hours per day coming here and going back home.” (S1) “Time consuming, we don’t have enough time to go back home and study…” (S2, S5)
Mental health	Ability to cope with stressful events by being mentally well.	PHYSICAL: “I myself have experienced a lot of anxiety issues … because of this learning system.” (S8) “I was admitted to the hospital so many times during my stress and anxiety…” (S9) BLENDED: “At home you will get the support of your family.” (S5)
Hectic	A state of daily incessant activities owing to a fully packed schedule with on-campus teaching 6 days a week.	“Social life is a bit hectic nowadays, as time is very limited.” (S1) “6 days a week does get a lot hectic, especially with 8-4 classes with posting.” (S6)
Manageable	A method that is feasible and easy for the student to deal with.	ONLINE: “Theory classes are manageable online.” (S5) BLENDED: “It will be easier to manage- learn more in a lesser amount of time.” (S6)
Hesitant	A state of reluctance to do a particular action while attending online classes.	“Students might be hesitant to unmute and question online.” (S5, S10)
Life balance	The ability of the students to juggle both their studies along their personal and social life.	IN PHYSICAL: “With the system that we currently have, it is difficult to have a good balance.” (S10) BLENDED: “It will be much better.” (S5, S10)
Self-directed learning	A study approach whereby the students take the initiative and learn the topic by themselves.	IN PHYSICAL: “I have always done that since I joined medical school.” (S7) “Quite difficult to do, due to the lack of time. I have been struggling.” (S2)
Efficient	A quality of being able to complete a task or lecture with minimal effort and time wasted.	ONLINE: “Often, we would be having wifi issues, had to wait before rejoining and took attendance. It wasn’t the most efficient, time productive lecture.” BLENDED: “Blended learning is more efficient and effective. We will be more on time with our syllabus.” (S2)
Efforts	An action of hard work and dedication towards achieving a goal.	ONLINE: “Teachers should put more effort into the presentation and PowerPoint.” (S7)
Personal time	A time for the student only, for activities which are not related to work or study, and is distinctive for each student.	BLENDED: “We will have more time for our family.” (S5) “It will be easier and we will have more time for ourselves.” (S6)
Time-saving	A quality where time usually spent on an activity, such as traveling to school, is reduced, and less time is wasted.	BLENDED: “We will save a lot of time compared to in-person classes.” (S2)
Class material availability	The idea of having online access to class notes and PowerPoint online, either before or after the class, in a blended learning system.	“Advantageous, as we will not waste time copying.” (S1) “Good idea, as we are able to prepare, go back and read, see what you don’t know. It is beneficial.” (S2) "Disadvantageous, as students will not follow the class since the material is already available. Also, there are many things the teacher says which are not in the PowerPoint.” (S10)
Revision	The process of going through the notes again to better understand the content and update it.	ONLINE: “I have more time to study and keep up.” (S7) BLENDED: “More time for revision.” (S5)
Active learning	A study approach, whereby students learn by triggering their thought process and engaging fully in class discussions.	IN PHYSICAL: “In face-to-face classes, we learn much more by asking questions.” (S5) “We have to read the PowerPoint, listen to the teacher, at the same time write the notes and this is why you cannot grasp a lot in the class.” (S10)
Interaction	An act of communication with or a reaction to the teacher, through which a relationship is established with the student.	ONLINE: “We didn’t have enough personal relationships with the teachers and we couldn’t ask questions.” (S5) “It is more weird/difficult to discuss or ask questions online.” (S2) IN PHYSICAL: “It’s very important to me, i feel that’s what we lack a lot here.” (S11) BLENDED: “Student- teacher interaction will not be affected as there will still partly be in physical classes.” (S5, S10)
Better approach	A more conventional study design, with superior learning and teaching techniques, as compared to what is currently in place.	BLENDED: “I can manage what I know and what I don’t know better. It will be easier and students will perform better.” (S6)
Sedentary lifestyle	The lack of physical activity and the increased number of hours sitting still which is promoted by online classes.	“Sitting too much and not walking will lead to morbidity for me.” (S4)
Physical well-being	Maintaining a healthy body through proper nutrition and physical activity which allows the most to be done on a day to day basis.	Physical: “Physical health not that much.” (S10) “I’ve let go of myself a lot, i was much more active , more sportive, much more thinner…” (S11) “It gets really daunting at times, especially when it is exam season.” (S6) Blended: “More time for myself, regular exercising, taking care of my diet.” (S6) “It will improve my sleep, it will improve my physical health definitely…” (S8)
Knowledge & confidence	In a blended system, knowledge and skills are acquired through visual based learning and simulation, which will allow students to be more comfortable in clinical setup.	“What I feel is missing here is the fact that we don’t have visual learning. I can know my theory well but if you put me in a clinical setting i will get confused.” (S7)
Burn out	The feeling of being overwhelmed resulting from constant mental and physical stress with the traditional system.	“No proper weekend, by the time exams come we already so burn out…” (S11) “We don’t have time to rest even after our professional exams and this is not good as the mind should be given a rest.” (S5) “We don’t have enough holidays.” (S5)
Internet connection	Issues with internet access that may be faced during online classes.	“There were a lot of issue with the audio and the video…” (S9)
Predominant mood	The state of mind an individual is most frequently in.	“Coming in the morning to college, with the mindset of ‘I don’t wanna be here, I am tired’ it automatically sets you up for not paying attention and having a bad day…” (S11)
Technological era	Rapid evolution in the technological field and new advancements that can be used to our convenience.	“We have so many resources, so much of data and so much of innovation to use..” (S11) “... the world is moving forward, as technology is advancing we should use it for our own advantage… if we look at any first world countries a lot of their lectures are held online… the first world countries and western world are still moving ahead.” (S11)

## Discussion

Quantitative domain

The quantitative aspect had an overall response rate of 81.14%. It is evident that students noted both the negatives and advantages of online and traditional lectures, specifically highlighting what would be useful and what challenges they faced. Additionally, 96.1% of the students who participated in the study had been exposed to the online method of teaching, and 67.6% of students indicated that they were in favor of having a mixture between online and traditional didactic lectures. This is supported by a study conducted by Wieling et al., who noted that different students gained different advantages from didactic and online lectures and that students who preferred the online modality produced the same results as those who attended didactic lectures, proving that they are equally as efficacious if used productively [10].

Online methods of teaching use various applications to connect and conduct these lessons; however, with Mauritius being a developing nation and with telecommunications being used in higher quantities due to the shift to online modes, students found difficulty with some of the applications. The major issues identified with the online lectures were platform and technological issues, with 26.2% of males and 19.8% of females identifying this as the major drawback to the online lectures. This sentiment is congruent with a study based on bandwidth and the perspective of online teaching performed by Wu et al., who depicted that the relationship between a good internet connection and how students perceived online learning was proportional [11]. A further study by Barut et al. identified the various trends of studying among Turkish medical students. They found that first-year students relied more heavily on Atlases and textbooks as opposed to their third-year counterparts, who preferred lecture notes. This finding is contradictory to that of this paper because, in Mauritius, online lectures affected the more senior students more adversely due to the reduced hands-on exposure in the clinical subjects, although marginally [12].

Additionally, 29.5% of male students and 34.6% of female students clearly noted that class engagement is the most common area that needs to be improved in order to make online lectures more viable. A portion (41.9%) of Indian students agreed that engagement within online lectures can be improved, whereas only 17.3% of Mauritian students agreed with the notion that online class engagement should be improved. Chwiłkowska-Kubala et al. noted that student engagement and active participation in online lectures are directly proportional to students’ satisfaction [13]. Online and distance education does not lend itself to open and active engagement to the degree that traditional didactic education does, and this poses one of the greatest challenges to education in the online space [14].

Even though a dichotomy of students exists between preclinical and clinical phases of medical school, 92.6% of male and 91.8% of female students agreed that online lectures could optimize their studying if better built into their study schedule. This finding is significant as students in the clinical phase rely more heavily on a hands-on approach; however, students still believe that online lectures have a positive and productive role in their education if effectively built into their study schedule. Additionally, 69.1% of females and 65.6% of males preferred a mixture of didactic and online lectures, 77.8% of Mauritian students, 73.9% of South Africans, and 61.7% of Indian students agreed with this sentiment. The ability of online lectures to work harmoniously with that of didactic lectures and be useful was strongly supported by a study performed by Simoniva et al. [15]. This showed that teachers who designed the program to be taught online and were trained in such lecture delivery produced improved overall results [15].

The overall response from students showed that a balance between online and traditional didactic lectures would be most beneficial to their learning. Students very clearly weighed up the drawbacks experienced in both online and didactic lectures while effectively highlighting the advantages that they noticed in both. The overarching finding and sentiment noted is that students require online methods to be effectively used as a tool and to be part of the traditional learning environment, creating a blended learning style of teaching that will utilize the advantages of both traditional and online methods to ensure the optimization of individual students' education. This should also be tempered with the fact that different students learn, understand, and retain knowledge in different ways, which can significantly influence their motivation and ability to absorb information. Visual learners grasp concepts more effectively through images, graphs, and diagrams, whereas auditory learners learn best through listening and speaking, while kinaesthetic learners benefit most from hands-on experiences and physical activities. Recognizing and catering to each student's unique learning preferences can greatly enhance their overall learning experience. The ability of online style lectures to cater to both the visual/auditory learners and allow the student the freedom to move while absorbing knowledge (in the case of kinaesthetic learners) may aid in being accepted by a greater proportion of students [16].

Qualitative domain

From the qualitative study carried out among medical students in SSR Medical College in Mauritius, through an inductive analysis, three major themes were formulated from the perception of their learning experience during the COVID-19 pandemic. These were in accordance with the various types of learning systems that they had experienced during that period: the online learning system, physical learning system, and blended learning system. Each theme was further broken down into specific categories - emotional, academic, and related variables - through which corresponding codes were sorted.

The thematic codes generated from each theme clearly reflected that each study design has advantages and disadvantages, and even the blended system is no exception thereto [17]. This study aims to not only share the perception of medical students toward web-based learning but also toward the traditional and blended learning systems through a thorough thematic analysis. The findings of this study shed light on the students’ perceptions while simultaneously carrying out an analysis of each learning system.

As the COVID-19 pandemic dragged on from March 2020 and showed no signs of relenting in the next few months, it was a clear indicator that restarting teaching through conventional methods might not be the solution to prevent students from lagging behind [18]. The best interests of the students at that point in time were in the unconventional teaching methods only. The COVID-19 full-blown pandemic left no option but for an online learning system to be adopted for a vast part of the MBBS curriculum for the very first time, not only in Mauritius but across the globe [19]. No alternative was used by students to describe the reason why internet-based learning was introduced in the first place.

But et al. described the physical learning system as one where students follow didactic lectures, read handbooks, and prepare handwritten notes [19]. Since physical learning previously formed the core of medical studies in Mauritius, some believed this form of lecture delivery to be better, as a student stated that “teachers can explain more to us and can see who is following” [18,19]. There was more discipline with in-person classes, but on the flip side to this, attending all classes was more time-consuming and made their lives more hectic since they had to be present at the institution for long hours [5]. For many, their predominant mood for attending classes the traditional way 6/7 was expressed by stating “I am tired” and admitting that this affected their learning capacity [5]. They claimed this system to be exhausting, causing them to suffer from burnout, which was supported by the findings in the study carried out by Al-Jehani et al. [20]. In the strictly in-person learning system, students shared that they could not have a good work/study-life balance. Students had conflicting views in regard to the process of active learning with the traditional teaching system as some stated that “in face-to-face classes, we learn much more by asking questions,” while others could not grasp enough, stating “we have to read the PowerPoint, listen to the teacher and at the same time write the notes and this is why you cannot grasp a lot in the class” [21]. Similar views were found when students were asked about their ability to do self-directed learning, a concept being highly encouraged by professors. With the traditional learning system, a few students found that they were able to do so with this system, while others struggled owing to a lack of time to do so [22,23].

As stated by But et al. [19], the online learning system consists of students utilizing resources such as online learning platforms, e-books, and multimedia, among other facilities, through their electronic devices. Today, we live in a technology era [24]. Learning through virtual platforms, such as Zoom and Webex, when in-person classes had stopped for safety reasons during the pandemic, was an eye-opening experience [25]. Students have shared how they appreciated the flexibility provided by this system. They had found it to be manageable even with their bulky syllabus during that period. They claimed to have more time at hand to do their revision and keep up with their studies [26]. However, they had conflicting views in regard to the class material. Some believed that it was “a good idea, as we are able to prepare, go back and read, see what you don’t know,” while others thought that idea to be “disadvantageous, as students will not follow the class since the material is already available” [26].

Just as with every other system, drawbacks from this online learning system were not spared. Many expressed their concerns over its further contribution to a sedentary lifestyle. The full-fledged online classes made students' “lives more boring than they already are” and only fuelled their laziness [25]. The fact that it was stressful for some as they were hesitant to ask questions during online classes is consistent with a study conducted by Nilaad et al. [26]. They did not always find it efficient, as they faced a lot of connectivity issues and wasted time logging into every online class [24]. Some students described the Zoom-/Webex-based learning as difficult because the educators were not all techno-savvy and most used only PowerPoint presentations for the lectures [25,26]. Many agreed that educators should put in more efforts to prepare for online classes and that there was, in fact, room for improvement in the quality of the classes conducted [25].

In regard to the effectiveness of face-to-face learning as compared to learning through virtual platforms only, there were contrasting reviews among the students because some felt more productive at home and others on campus [27]. Some expressed mixed feelings toward the online learning systems. It was right for a few things, as mentioned above, but also inappropriate when it came to clinical and practical aspects of medicine. Experience gained by medical students in this field is of paramount importance. Students believed that “we really need to go to the hospital, interact with the patients, and the environment to have more hands-on experience in postings,” and this further concurs with the findings of ​Berberoglu et al. [23]. This was a critical defect in this system that students have suffered from in the strictly online classes during the COVID-19 pandemic. Hence, medical students were convinced that there should be judicious use of the virtual platforms available and use them only in specific theory classes [24].

Makhdoom et al. described blended learning as a method of combining electronic and face-to-face learning [17]. From the study conducted, 90.9% have shown interest in integrating this system into their daily schedule. The remaining 9.1% were unconvinced only due to the lack of infrastructure and training of both educators and students. Moreover, 100% agreed that working hand-in-hand with visual-based simulations and hands-on practice on cadavers would allow them to have better knowledge and confidence in this field [18]. With blended learning, there is the opportunity to capitalize on the best from both worlds, that is, taking the good from both the in-physical and online learning systems and minimizing the disadvantages [24]. Students believe that this mixed system would be more manageable and less exhausting. As further validated by the evidence from the study undertaken by Vallée, et al., a blended learning system will be more effective, efficient, and time-saving [5]. Students would have more personal time, during which they can continue their revision and take better care of their mental health [18]. With the blended system, students were sure of an expected improvement in their physical well-being because one of them mentioned, “it will improve my sleep, it will definitely improve my physical health.” They believe that the interaction between student and teacher will also not be very much affected as they will not be completely cut off from them [18].

From the study conducted, it has been shown that most of the students have had the experience of both worlds - completely in -physical and completely online classes and, from that, know their pros and consHence, they came up with the idea that the blended system would be a better approach to their medical studies. Overall, the blended learning system would lead to a net improvement in the student life balance [23]. In today’s modern era, where artificial intelligence is the new vogue, it forms an inseparable aspect of our day-to-day lives, including our studies. It can be incorporated in the curriculum by including 3D visual-based simulations of the anatomy of the human body and different medical procedures, which would contribute toward a greater understanding of the subject as substantiated by the study of But et al. [19].

A good timetable accommodating both types of classes would marginally improve students’ lives and allow teaching to be more effective and efficient [28]. Implementation of a blended system, which is well-organized and adequately balanced with online and in-person classes, should be tailor-made to the requirements of each course and the infrastructure available at the level of the institution, as well as the students. As stated by But et al., quality medical education is the foundation for a successful medical system, and this can only be improved by constantly evolving with recent advancements made [19]. A strategic and well-maintained blended system for the medical course can make a significant positive impact on future doctors of the medical system and should hence be further explored [18]. Moreover, with the emerging cutting-edge technology available, the blended system can even be ameliorated by including facilities such as virtual dissection tables and augmented reality, among others, to further improve the teaching methods, which would consequently yield better student learning outcomes [28].

Strengths of the study

This mixed methods study, with a high response rate of 81.14% on medical students' perspectives on physical, online, and hybrid learning modalities, is the first known such study to take place on the tropical island of Mauritius and gives us an insight into a very niche area, which can be further extrapolated in both developed and underdeveloped nations. This study provided an in-depth evaluation of the three themes of the study - physical learning system, online learning system, and blended learning system. Most of the students participating had experienced at least two different types of learning systems. Hence, the study also provided an important insight into the students’ perception of each learning method, valuable feedback that can help improve the system in the future. The study included a large sample size of 568 for the quantitative domain and a good mix of both national (Mauritian) and international students (Indian and South African). Both males and females were included, and students participating in the study were from the first to the final year of the MBBS course. Hence, there was good diversity in the study population. The study provided good clarity on all three learning systems and elaborated on the strengths and weaknesses of each, from the perspective of a medical student. The shortcomings of the traditional learning system were not only highlighted, but a plausible solution, agreeable to most students, was also proposed using the evidence from the data analyzed.

Limitations 

The participants included in the study were from a single medical institution in Mauritius. A multicentric mixed-methods research with a higher sample size is required in the future to find out the views of view from various fields on hybrid, online, and physical classes all over Mauritius. Furthermore, students across various fields, not only medicine, can be included in a larger study in the future. Some students might have shaped their answers so as to be in line with the novel learning methods. This might cause a discrepancy in the validity of their answers as per their genuine perceptions. Few students in the quantitative study had not previously been exposed to online learning, and most students, though in favour of the blended system, had not had the opportunity to experience it fully.

## Conclusions

When it comes to learning and education, one size does not fit all. Every student has their own learning style, each with a unique mix of visual, aural, reading/writing, or kinesthetic styles. A strictly online or physical learning system would not align with the best interests of all students; hence, a middle ground, which can effectively bridge the gap, has to be found. Overall, students were positive that the hybrid model of teaching and learning is the best option to optimize their education in a wholesome and holistic manner and is better suited to overcome these challenges. This study is therefore pertinent and contributes to the dearth of data that exists with regard to the views of students on hybrid teaching in Mauritius and provides valuable insights into the difficulties faced by students, as well as the advantages noted through the study. The novelty of this study is evident, and its practical implications stretch far beyond the lockdown and COVID-19. However, its findings, particularly, surrounding the reduction in the loss of class times during tropical storms and hurricanes, are invaluable. Although specific and unique to Mauritius, this blend of learning can reduce the loss of education in more common unforeseen circumstances, such as hurricanes. The hybrid model of teaching caters to a wider audience of students and helps create a balanced mental and physical educational experience, resulting in the best outcome for students in their studies by capitalizing on the advantages of both didactic and online lectures to produce a method of education with more benefits than detractors to ultimately produce better and more capable medical doctors.

## Appendices

Annexure 1

Questionnaire

Instructions to the participants: 

There are some questions about “Medical students' perspectives on Physical, Online and Hybrid learning modalities: A Mixed Methods Study from a Medical School in Mauritius” comprising of 2 sections A, B read it carefully, thoroughly, understand the questions and answer them accordingly. 

Please note that your answers are completely anonymous and that the data collected for this study will be used solely for the study and after which it will be deleted. You reserve the right to withdraw your data from the study at any point in duration of the studies progress.

A. Demographic details:

1. What is your Age (in years)? ____ years

2. Nationality:

i. Indian

ii. Mauritian

iii. South African

iv. Others

3. What is your Gender?

i. Male

ii. Female

4. What is your Religion?

i. Hindu

ii. Muslim

iii. Christian

iv. Atheist

5. What MBBS professional are you currently in?

i. 1st MBBS

ii. 2nd MBBS

iii. Final Part 1

iv. Final Part 2

6. What is the level of study?

i. Preclinical

ii. Clinical

B. Quantitative domain

Hybrid, Online classes vs Physical didactic lectures

1. Have you experienced any online lectures/classes (during COVID-19, rainy days or for any reason)?

i. Yes

ii. No

2. If Yes for Q.1., did you feel that the online lectures affected your education negatively?

3. Which do you prefer? Preference for teaching and learning methods:

i. Blended learning (online and in person)

ii. Didactic lecture (in person)

iii. Online

4. What improvements can be made to online lectures?

i. Better preparation

ii. Class engagement

iii. Content

iv. Internet

v. Timing

5. Can online lectures, when better built into a timetable, better optimise your studying?

i. Yes

ii. No

6. How often would online lectures be acceptable and useful?

i. More than once weekly

ii. Once weekly

iii. Only rainy days

iv. Never

7. What was the duration of your online lectures?

i. Less than 1 month

ii. 1 month

iii. 3 months

iv. 4 months

v. 6 months

8. Weaknesses of online lectures?

i. Distractions

ii. Lack of accountability

iii. Lack of interaction

iv. Platforms used

v. Lack of student attention

9. Strength of online lectures?

i. Ease of accessibility and timing

ii. Enhanced lecture emphasis

iii. More self-directed study time

10. Can hybrid lectures be made part of the syllabus?

i. Yes

ii. No

11. What online platform was used to conduct the lectures?

i. Google Meet

ii. Microsoft Teams

iii. Webex

iv. Zoom

12. Online lectures affected you negatively?

i. Yes

ii. No

C. Qualitative domain:

1. How was the online teaching experience? What are your feelings towards online classes? 

2. Can you tell us a bit about your experience with strictly on-campus learning? What are the advantages of being physically present in all of the classes and postings? 

3. Any issue with this system that we currently have and any suggestion to solve the difficulties that you face while learning in physical 6 days a week?

4. How would you describe your student life balance/ social life along with studying, with strictly on-campus learning? 

5. Would you prefer if online classes were incorporated into the daily schedule?

Yes/No

6. Why do you say so?

7. What do you understand by a blended learning system? What is your point of view on this?

8. Mixture of both online and in physical classes/ different learning styles & materials. What is your point of view on this?

9. How would your student life balance change with this system? 

10. How about student-teacher interaction? Is it important to you?

11. Do you take notes in class?

Yes/No

12. In a scenario of a blended system where you could access all of the class materials online, how would it be to your advantage or disadvantage? In which setting are you able to gain the maximum knowledge? 

13. Nowadays, self-directed learning by students is being encouraged. How feasible do you think this is with strictly on-campus learning? 

14. In a blended system, how would your ability to keep up with the pace at which the syllabus is being covered be affected? 

15. About your preparations for class tests, how would they change with a blended system? 

16. What are your views on a blended education system, with regards to physical, practical/clinical hours? 

17. In a blended learning system, where you could experience visual-based simulations, in which way would it change your anatomic conceptualization and clinical skills?

18. Currently, are you able to take care of both your physical and mental health?

19. If you had a choice between a blended learning system and a strictly on-campus/in physical system, which one would you prefer and why?

Annexure 2

Informed Consent Form

Type of research intervention

This research will involve completing an online questionnaire/survey.

Participant selection

We are inviting all students of SSR Medical College, Mauritius to participate in the study

Voluntary participation

Your participation in this research is entirely voluntary. It is your choice whether to participate or not. You may change your mind at a later stage and stop participating even if you agreed to do so earlier.

Duration 

It will take 5 minutes to complete the process.

Confidentiality

The information collected via this survey will be kept confidential. All Information about yourself that will be collected will be stored safely, and no one but the researchers will have access to the data.

Right to refuse or withdraw

You do not have to take part in this research if you do not wish to do. Refusing to participate will not affect you in any way. You may stop participating in the research at any point in time. It is your choice, and all of your rights will still be respected and upheld.

Whom to contact

If you have any questions at any stage before, during or after the completion of the study, please do not hesitate to contact us. Their contact details are provided below.

Dr. Indrajit Banerjee, Professor, SSR Medical College, Mauritius

Email: indrajit18@gmail.com; Phone: +23058832236

1. I understand that my name and initials will not be published, and researchers will keep my anonymity. 2. I voluntarily agree to participate in this research study. 3. I understand that all information I provide for this study will be treated confidentially. 4. I understand that the material may be published in a journal, medical books, web website, or other forms of publication. 5. I understand that I am free to contact any of the people involved in the research to seek further clarification and information.

I have read the above information. I understand and agree to the terms and conditions described above and give my consent to participate in this study.
